# The Role of Trait and State Absorption in the Enjoyment of Music

**DOI:** 10.1371/journal.pone.0164029

**Published:** 2016-11-09

**Authors:** Sarah E. Hall, Emery Schubert, Sarah J. Wilson

**Affiliations:** 1 Melbourne School of Psychological Sciences, University of Melbourne, Parkville, Victoria, Australia; 2 School of the Arts and Media, University of New South Wales, Sydney, New South Wales, Australia; University of Zurich, SWITZERLAND

## Abstract

Little is known about the role of state versus trait characteristics on our enjoyment of music. The aim of this study was to investigate the influence of state and trait absorption upon preference for music, particularly preference for music that evokes negative emotions. The sample consisted of 128 participants who were asked to listen to two pieces of self-selected music and rate the music on variables including preference and felt and expressed emotions. Participants completed a brief measure of state absorption after listening to each piece, and a trait absorption inventory. State absorption was strongly positively correlated with music preference, whereas trait absorption was not. Trait absorption was related to preference for negative emotions in music, with chi-square analyses demonstrating greater enjoyment of negative emotions in music among individuals with high trait absorption. This is the first study to show that state and trait absorption have separable and distinct effects on a listener’s music experience, with state characteristics impacting music enjoyment in the moment, and trait characteristics influencing music preference based on its emotional content.

## Introduction

Does personality influence the kind of music people like? Research suggests that people with an openness to new experiences tend to prefer music that is ‘complex’, whilst people who are extroverts tend to like music that is energetic and rhythmic [1]. Contrasting with this view is the perspective that event driven, psychological and cultural factors influence preference formation more than personality [2]. That is, preference is primarily shaped by the stimuli to which an individual is exposed. Empirically there have been no explicit tests of whether our experience of music is mediated by trait characteristics, such as long-term behavioural habits, personality or disposition, as compared to state characteristics that are incident or event-based and occur in the moment. The present study aimed to address this question by examining determinants of musical preference. Specifically, do state and trait characteristics influence musical preference differently?

One methodological problem in disentangling the primacy of state versus trait in preference research is to find a measure of personality that is compatible with a measure of one’s response to an event (such as listening to a piece of music). Some personality traits do not always sensibly translate to specific experiences; for example, it would be unusual to feel extroverted in response to a piece of music. One exception to this is absorption, which is linked to the personality trait of openness to experience. Herbert [3] found that engagement with music is an important way of inducing absorption, making the study of music and absorption particularly appealing. Thus, in this study we investigated the relationships between music preference and trait versus state absorption.

### State versus trait absorption

Tellegen and Atkinson [4] proposed the most enduring definition of trait absorption as “a disposition for having episodes of ‘total’ attention that fully engage one's representational (i.e., perceptual, enactive, imaginative, and ideational) resources” ([4], p. 268). They subsequently based their metric of trait absorption on a personality dimension labelled “Openness to Absorbing and Self-Altering Experience”.

It has been argued that “causally, the trait of absorption is […] a proximate antecedent of the state of absorption” ([5], p. 668). However, other factors such as expertise, environment and mood may also mediate state absorption. For example, a highly skilled painter may enter into states of absorption during the act of painting more frequently than when performing other, non-painting activities. During the performance of an activity like painting, playing sport or music, the analogue for state absorption is ‘flow’ [6]. This most typically occurs when there is a match between the skill of the performer and the challenge requirements of the activity [6, 7].

The contribution of state versus trait absorption to the experience of an activity is not clear-cut [3, 8]. For instance, it might be easy to induce state absorption in a laboratory setting in an individual high on trait absorption, whereas in a more familiar, comfortable environment, state absorption might be induced in a wider population [9]. A person’s antecedent state or mood, such as being in a negative mood or in pain, may also prompt an individual to seek out states of absorption as a form of escape and distraction, regardless of underlying trait absorption [10].

Herbert [3] investigated the relationship between state and trait absorption by applying an Interpretative Phenomenological Analysis to experiential reports of state absorption, and correlating these with quantitative ratings of trait absorption. No relationship was identified between state and trait absorption, leading Herbert to suggest that trait absorption “may simply not tap all experiential aspects of absorption” (p. 58). Arguably, however, a qualitative approach to coding absorbing experiences may preclude adequate comparison with a psychometric scale of trait absorption. One solution, albeit at the expense of losing the kinds of experiences in which absorption occurs, is to adopt an equally reductive, quantitative measure of state absorption. For example, Pekala [11] included a scale of state absorption in his Phenomenology of Consciousness Inventory (PCI) that measures “how intensely involved in the object of attention the subject is” (p. 145) through direct probing of the subjective experience of being absorbed.

### Trait absorption and the enjoyment of negative emotion in music

Evidence suggests that people who are high on trait absorption enjoy various forms of art [12]. For example, trait absorption scores are correlated with liking complex (classical in comparison to rock) music [13], a finding that is consistent with the similar trait of openness to experience [4, 14, 15]. In reviewing the literature, however, Roche and McConkey [8] suggested that rather than complexity, the relationship between absorption and liking may be mediated by “personal involvement of a cognitive or an emotional kind with the attentional object” (p. 94). This idea is supported by the work of Kreutz and colleagues [9] who collected trait absorption data and emotion ratings induced by 25 excerpts of music. Participants high in trait absorption had more intense emotional reactions in response to the music than those with low trait absorption [16]. Moreover, trait absorption was positively correlated with arousal induced by the music, with the highest correlation for music that induced sadness. An explicit test of the relationship between trait absorption and negative emotion was reported by Garrido and Schubert [17]. They collected trait absorption data and asked participants to rate the extent to which they liked sad music. Regression analysis showed a strong linear relationship between trait absorption and liking sad music.

The evocation of sadness in an individual that is also enjoyed has been a topic of ongoing research debate. While evidence supports that grief, tragedy, sadness and other negative emotions are enjoyed in all art-forms [18], researchers remain sceptical (for a review, see [19]). This scepticism stems from the idea that negative emotions, such as sadness and grief, are associated with withdrawal and aversion under normal circumstances. Thus, there must be some extenuating circumstance to explain the concomitant pleasure response, and as such, ‘real’ negative emotion is not experienced [19, 20].

A less sceptical view suggests that personality traits, such as absorption and empathy, explain the paradoxical attraction [17]. Schubert [21] proposed that negative emotions can be enjoyed in an aesthetic music context because displeasure is ‘switched off’ under these circumstances, allowing negative emotions to become activated without their unpleasant, painful effects. This ability to disconnect from the unpleasant aspects of the experience is likely to be enhanced in individuals high on trait absorption, since absorption represents a disconnection from one’s surroundings and immersion in internal thoughts, processes and mental imagery. According to this theory, it should be possible to disconnect from, and thus enjoy, a range of negative emotions in music. Despite this, previous research has tended to focus almost exclusively on sadness.

### Gap Across Emotion Loci

A relatively new aspect of music preference investigation is the idea that music is liked more if the emotion expressed by the music (external locus of emotion) is well matched with the emotion evoked (internal locus of emotion) by the music [22, 23]. For example, people like sad music more if the music not only expresses sadness, but also invokes feelings of sadness in the listener ([24], see also [25], p. 258 and his concept of contagion). The few studies that have investigated this aspect of preference have replicated the finding (for a review, see [22]), but they have focused mainly on ratings of valence and arousal because these are thought to be the main underlying dimensions of emotion particularly since the work of Russell [26].

If matching between emotion loci (felt by the listener versus expressed by the music) is an important part of music preference, it seems that a wide range of emotion variables should be well matched, not just the traditional dimensions of valence and arousal. Since strong experiences are phenomenologically important in music listening [27, 28], emotional strength will also be an important part of that experience, both in terms of the strength of the emotion felt and the strength of the emotion expressed by the music. Emotional strength is an aspect of emotion not explicitly included in Russell’s circumplex model, but because of its important link with strong experiences in music [27], it may be related to the psychological construct of absorption.

Thus, in this study we aimed to investigate the impact of both state and trait absorption upon preference for music, particularly our preference for music that evokes negative emotions. The literature suggests that trait absorption might be the critical arbiter of absorption with a stimulus, but we have found no research explicitly testing the additional role of state absorption. The idea of trait absorption being an antecedent of state absorption suggests that both trait and state absorption will predict enjoyment of music, and in particular the paradoxical case of music that evokes negative emotions.

## Method

### Participants

128 participants (66 females) took part in the study (mean age = 21.1 years, *SD* = 2.92, range 18–34 years). The cohort reported having played a musical instrument for an average of 6.54 years (*SD* = 5.19, range 0–20 years).

### Stimuli

Participants each selected their own pieces of music to use as stimuli. We used self-selected music as it is considered to be most effective in eliciting strong emotional responses [29]. A point of departure in the present study, however, was the type of music that participants were asked to select. Typically preference studies ask participants to select ‘loved’ versus ‘hated’ music (e.g. see [30, 31, 32]), however this can lead to a disproportionate number of extreme scores with the expected preference producing ceiling or floor effects. Since the purpose of this study was to elicit absorption and strong emotional responses, we asked participants to choose music they found ‘powerful’ or ‘boring’. We anticipated that these selections would produce a range of emotional responses and preference scores. We expected overall positive preference responses for powerful music based on Rickard [28], who found that emotionally arousing, powerful music was more liked than three other categories of music (referred to as ‘relaxing’, ‘arousing-but not powerful’ and ‘film’ music). Furthermore, one of the reasons music is rated as boring is because it is heard too frequently [33]. Since the prevalence of frequently heard music is high in Western cultures [34], the task of selecting boring music should be easier than selecting explicitly ‘hated’ music, which individuals may find hard to identify. There were no constraints placed on the duration, genre, or style of the pieces that participants were permitted to select.

### Measures

To assess the contributions of state and trait absorption to enjoyment, it was important to include a range of variables implicated in preference in addition to absorption. Based on the work of Schubert [32], we developed a listening appraisal questionnaire, which included the explicit rating of negative felt emotion.

#### Listening appraisal

The listening appraisal questionnaire collected subjective ratings of preference, emotion, familiarity and quality of the self-selected powerful and boring pieces of music through 19 items per piece (see Table 1). Items were rated on an 11-point Likert-type scale that ranged from 0 (‘strongly disagree’) to 10 (‘strongly agree’), with the mid-point of 5 labelled ‘neither’. Sample words for the emotion items were taken from three sources of words mapped onto emotion spaces [35–37] and rated using unipolar scales (see [32] for a detailed explanation). Although bipolar scales are used more typically in the literature (e.g., excited-sleepy; positive-negative), the use of a single bipolar scale for valence excludes the scenario where a listener experiences both positive and negative emotions at the same time, as is the case for liking sad music [38]. Since familiarity and the perceived quality of the music also impacts upon preference [32], items rating these constructs were included.

**Table 1:** Means and standard deviations for listening appraisal items and absorption measures.

Construct	Item/Scale	*M (SD)*	
		Boring piece	Powerful piece
**Preference**	I love this piece	2.73 (2.61)	8.97 (1.79)
**Quality**	This was good quality music	4.96 (3.05)	8.96 (1.49)
**Familiarity**	To me the music is familiar	6.75 (3.12)	9.12 (1.61)
**Emotions (internal locus)**	I felt strong emotions	2.90 (2.84)	8.28 (1.89)
	I felt positive emotions (e.g., happiness, serenity)	2.89 (2.58)	6.58 (2.85)
	I felt active emotions (e.g., excitement, rage)	3.22 (2.90)	5.36 (3.25)
	I felt dominant emotions (e.g., anger, determination)	2.71 (2.70)	4.39 (3.23)
	I felt no emotion	5.36 (3.17)	1.00 (1.92)
	I felt negative emotions (e.g. sad, angry)	3.40 (3.07)	3.16 (3.34)
	I felt sleepy emotions (e.g. calm, bored)	4.88 (3.24)	2.88 (2.97)
	I felt submissive emotions (e.g. fearful, surprised)	1.85 (2.21)	2.33 (2.80)
**Emotions (external locus)**	The music is emotional	3.76 (2.91)	8.3 (1.772)
	The music expresses positive emotions (e.g., happy, serene)	4.51 (2.88)	6.21 (3.08)
	The music expresses active emotions (e.g., excited, enraged)	4.43 (2.82)	5.7 (3.27)
	The music expresses dominant emotions (e.g., angry, determined)	3.74 (2.95)	4.64 (3.36)
	The music expresses no emotion	3.25 (3.03)	0.73 (1.62)
	The music expresses negative emotions (e.g. sadness, anger)	3.46 (2.86)	4.21 (3.53)
	The music expresses sleepy emotions (e.g. calmness, boredom)	3.38 (3.21)	3.13 (3.00)
	The music expresses submissive emotions (e.g. fear, surprise)	2.45 (2.54)	3.17 (3.04)
**State Absorption**	PCI (Absorption)	6.13 (3.09)	9.42 (2.56)
**Trait Absorption**	MODTAS	66.27 (23.878)	

PCI = Phenomenology of Consciousness Inventory; MODTAS = Modified Tellegen Absorption scale

#### State absorption

State absorption was measured using the two item Absorption subdimension of the Phenomenology of Consciousness Inventory (PCI) reported by Pekala (11). Each item comprises two opposing statements separated by a 7-point Likert-type scale. Selecting a number closer to the left dipole (0) endorses the statement on the left, whereas selecting a number closer to the right dipole (6) endorses the statement on the right. The statement at each dipole for one of the items was: “I was continually distracted by extraneous impressions or events” (left dipole) and “I was not distracted, but was able to become completely absorbed in what I was experiencing” (right dipole). The response to this item was reversed. The statement at each dipole for the other item was “I was forever distracted and unable to concentrate on anything” (left dipole) versus “I was able to concentrate quite well and was not distracted” (right dipole). The state absorption score was obtained by summing the response of the two items. Higher scores on this subdimension reflect greater inward and absorbed attention, and have been shown to be positively correlated with trait absorption measured with the TAS [39]. The internal reliability of this subscale is high (.80) as measured using Chronbach’s alpha (α) [11].

#### Trait absorption

Trait absorption was measured using the modified version of the 34 item TAS [40]. The original scale was developed by Tellegen and Atkinson [4] as part of their project to identify the psychometric properties of hypnotisability. The scale was revised in 1982 [41], and has been modified by others. One commonly used version is the MODTAS (MODified Tellegen Absorption Scale) proposed by Jamieson [40]. The MODTAS employs a 5-point Likert-type scale (0 = never to 4 = very often) in place of the dichotomous true/false format of the TAS. This modified scale has been shown to have improved psychometric properties than the original scale, with a higher level of common variance and clearer factor structure (38). Participants are asked to record the frequency with which they have certain experiences (e.g. “I am greatly moved by eloquent or poetic language”). The MODTAS has been reported to have an internal reliability of α = .96 [42].

### Procedure

Participants were recruited from an undergraduate course that was opened for enrolment to music students and the wider student community at a university in Australia, in accordance with ethics requirements. This convenience sample was limited by the number of students enrolled in the course and the interest of those students in participating. The course was known to consist of some students who were studying for a degree in music (n = 25), ensuring that there would be a good spread of musical experience across the sample. The participants received course credit in return for their participation. They completed the study in their own time and were requested to find a quiet, private space and use a device with a good sound system (speakers or headphones). They were emailed a link to the experiment, which was presented via online survey software (Keysurvey, www.keysurvey.com).

Participants gave written consent to participant in the study before they were emailed the link. Written consent was provided by the participant after reading the Participant Information Sheet. The research was approved by the University of New South Wales Ethics Committee, approval HC13015.

After receiving information about the general nature of the study, participants were told that they would be instructed to listen to one powerful and one boring piece of music of their choice, with the proviso that each should be available through an online streaming service such as YouTube or Spotify if possible. The participants clicked a link to commence the study. They were then asked to listen to one of the two pieces (powerful or boring—order of the instruction selected by the survey software at random) according to the following procedure. Participants opened a new window on their web browser, and located the self-selected piece (e.g. via YouTube). They then copied the name, the performer/composer (as appropriate) and the url of the piece into the three corresponding fields of the survey window (name of piece, performer/composer, url). These data ensured that basic information about the piece was made available to the researchers to allow later identification of the selected pieces. They were then instructed to return to the window where the self-selected piece was loaded and start playing the piece. When participants commenced playing the piece they were asked to immediately hide the window in which the music was being streamed in case there were any distracting visual materials. After listening to each piece, they answered the listening appraisal items, followed by the state absorption items. After the two listening tasks participants completed the MODTAS. At the conclusion of the experiment, the participants entered sociodemographic information.

## Results

The results are organised into three sections: (1) data preparation and descriptive statistics, (2) enjoyment of negative emotions in music, and (3) the role of trait and state absorption, and of listening appraisal responses, in predicting music preference. This final section included an analysis of the contribution of negative felt emotions on preference as a function of trait absorption. SPSS version 22 (SPSS Inc., IBM, Chicago) was used for all statistical analyses.

### Data Preparation and Descriptive Statistics

Data for two participants were eliminated because they did not complete the survey, leaving N = 126. Investigation of potential order of stimulus presentation effects revealed no significant differences between listening appraisal items according to order of music listening (*p*s >.05). In this sample the MODTAS showed high internal reliability with α = .95.

The means and standard deviations for the listening appraisal and absorption variables are presented in Table 1. To assess preference for the musical stimuli, we examined response ratings for the item ‘I love this music’ for both the powerful and boring pieces of music. Overall, the powerful piece showed a high level of preference (*M* = 8.97, *SD* = 1.79), while the boring piece was associated with low preference (*M* = 2.75, *SD* = 2.60). Inspection of the distribution of preference scores showed that each piece elicited an appropriate spread of scores (range: 0–10). As expected, there was evidence of positive skew for the boring piece (skew = 0.951), and negative skew for the powerful piece (skew = -2.773). To mitigate this skewness and facilitate further analysis, we pooled the preference data for the boring and powerful pieces. These pooled scores were then divided into three different levels of preference: ratings of 0 (Low loved), ratings of 10 (High loved) and ratings in between these extremes (Mid loved).

### Enjoyment of Negative Emotion in Music

#### Can negative emotions be enjoyed?

Of the 68 participants who selected highly loved pieces, 24 (35.3%) reported high felt negative emotions (defined as greater than the scale midpoint of 5) whilst 49 (72.1%) selected reported low felt negative emotions (defined as ≤ 5). This provides evidence that listeners can have negative emotional experiences to music that they love. The result is supported by chi-square tests, which were significant when the expected value for the negative emotion cell count was set very low (count of 5 set as the expected value). Moreover, a significant difference occurred for all expected cell counts of high felt negative emotions, up to and including 18%, *X*^*2*^(1, *N* = 68) = 4.55, *p* = .033). This is consistent with previous research that has found that between 10% (Ladinig & Huron, submitted, cited by [24]) and 32% [21] of listeners choose loved music that makes them feel negative emotions.

**Fig 1:**
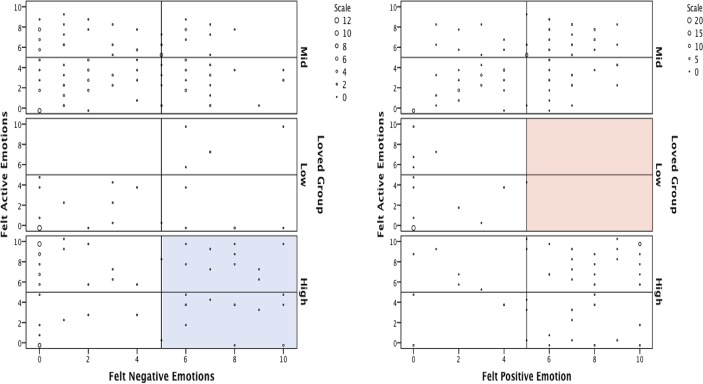
Density plots of felt negative or positive emotion against felt active emotion by Loved rating groups. Note that the blue region denotes the high felt negative emotion density cell for the High loved group (24 cases), and the red region denotes the ‘symmetric opposite’ high felt positive emotion density cell for the Low loved group (i.e. disliked) (0 cases). The comparison of these two regions demonstrates that the spread of negative emotions experienced in response to loved music is relatively large.

To explore this question further, we examined the spread of felt emotion scores for loved music using density plots (Fig 1), which are scatterplots that show the frequency of occurrence for each integer co-ordinate. These were generated in a two-dimensional emotion-space format, with negative or felt positive emotion ratings used to represent the x-axis ‘valence’, and felt active ratings used to represent the y-axis ‘arousal’ found on conventional emotion-space plots (see [26]). As shown in the cell highlighted blue in Fig 1, 24 participants reported experiencing felt negative emotion above the scale midpoint (>5) while listening to music that they loved (*M* = 8, *SD* = 1.53). In contrast, there are no scores present in the ‘symmetric opposite’ cell of positive felt emotion ratings (>5) for the low loved group (highlighted in red), meaning that none of the participants reported feeling positive emotions while listening to music that they disliked. This further supports the notion of enjoyment of negative emotions in music listening as a distinct phenomenon.

#### What kinds of negative emotions can be enjoyed?

To investigate whether negative emotions enjoyed in music are limited to low arousal emotions, such as sadness, we examined the vertical spread of scores along the y-axis (felt active emotions) within the high loved and high felt negative emotion group (blue cell). As can be seen in Fig 1, there was considerable variation in the degree to which the enjoyed felt negative emotions were rated as arousing (*M* = 5.3, *SD* = 3.31, range = 0–10). This suggests that individuals can report negative emotions with a relatively wide range of arousal across the Russell circumplex in response to music that they love. This is contrary to previous literature suggesting that low arousal negative emotions such as sadness are most prevalent.

### Absorption and Music Preference

#### The role of absorption in the enjoyment of negative emotions during music listening

To investigate whether trait and state absorption could explain enjoyment of negative emotion, we performed a median split of the trait and of the state absorption scores and ran chi-square tests of each against high and low negative felt emotions. The cross tabulation results for state and trait absorption against high versus low negative felt emotions are shown in Table 2. Expected counts reflect the counts that are expected if responses are distributed equally for each category. Counts of the high *trait* absorption group are proportionally higher for high felt negative emotions (count of 17) when compared to the number of participants in the low *trait* absorption group who reported high felt negative emotions (count of 7; *X*^2^(1, *N* = 68) = 4.03, *p* = .045). This demonstrates that individuals with high trait absorption were more likely to report high negative emotions while listening to loved music than individuals with low trait absorption. Counts for *state* absorption were statistically equivalent to chance in terms of their distribution for negatively valenced emotions for loved music (*X*^2^(1, *N* = 68) = 0.11, *p* = .736).

**Table 2:** The association of trait and state absorption with highly loved music.

			Felt Negative Emotions		
			Low (≤5)	High (>5)	Total
State Absorption	Low	Observed	6	4	10
		Expected	6.5	3.5	10
	High	Observed	38	20	58
		Expected	37.5	20.5	58
Trait Absorption	Low	Observed	24*	7*	31
		Expected	20.1	10.9	31
	High	Observed	20*	17*	37
		Expected	23.9	13.1	37
Total		Observed	44	24	68
		Expected	44	24	68

* *p* < .05 for chi-squared tests

Characteristics of liked music and the contribution of absorption. We next examined the extent to which state and trait absorption contributed to music preference more generally. Partial correlation analyses were performed using Spearman’s rho, pairing Loved ratings with either state or trait absorption scores, whilst partialing out the contribution of the other trait or state respectively. These analyses were performed for the powerful and boring pieces separately to ensure that there was a balanced contribution of state absorption data (trait absorption was constant for both pieces). For the powerful piece, state absorption correlated significantly with Loved ratings (*ρ*(TAS) = .34, *p* = .001) with trait absorption partialed out. However, trait absorption did not correlate with Loved ratings when state absorption was partialed out (*ρ(*ABS) = .159, *p* = .072). The pattern of findings was similar for the boring piece, with stronger correlations between Loved ratings and state compared to Loved ratings and trait absorption, although neither partial correlation reached statistical significance (state absorption *ρ*(TAS) = .152, *p* = .101, trait absorption *ρ*(ABS) = .012, *p* = .762). Overall, state absorption was related to music preference, whereas trait absorption was consistently not related to music preference.

Analyses were also run using the pooled Loved ratings for the powerful and boring pieces, as described earlier. Chi-square analyses were conducted for the three levels of Loved ratings (low, mid, and high) by trait absorption and by state absorption groups (each based on counts for high/low median split). A significant chi-square was found for state absorption (*X*^2^ (2, *N* = 227) = 30.22, *p* < .001) but not for trait absorption (*X*^2^ (2, *N* = 226) = 1.29, *p* = .525).

As Table 3 demonstrates, counts for the above median state (High) absorption group occurred proportionally more for loved music, while the low state absorption (Low) group had a proportionally lower observed count than expected for the highly loved piece of music. This suggests that participants who entered an absorbed state were more likely to rate their chosen piece of music as highly loved than participants who did not enter an absorbed state. This was not the case for trait absorption, meaning that there was no general propensity of a person with high trait absorption to give a rating of 10 for a loved piece any more than a person with low trait absorption. This analysis further supports the significance of state absorption over trait absorption in contributing to enjoyment of music in the moment.

**Table 3:** The association of trait and state absorption with loved rating groups.

			Loved Rating Group			Total
			Low	Mid	High	
Trait Absorption Group	Low	Count	17	68	31	116
		Expected Count	16.4	64.7	34.9	116.0
	High	Count	15	58	37	110
		Expected Count	15.6	61.3	33.1	110.0
Total		Count	32	126	68	226
		Expected Count	32.0	126.0	68.0	226.0
State Absorption Group	Low	Count	22	56	10*	88
		Expected Count	12.4	49.2	26.4	88.0
	High	Count	10	71	58*	139
		Expected Count	19.6	77.8	41.6	139.0
Total		Count	32	127	68	227
		Expected Count	32.0	127.0	68.0	227.0

* p < .05

Of the listening appraisal items, we wanted to determine those that made the greatest contribution to the liking of the music. We also wanted to examine the contribution of gap across emotion loci (GAEL) because of its explanation of preference response [22]. The GAEL score was calculated by taking the absolute difference of the respective felt and expressed ratings for each of the four emotion locus pairs (strength felt and expressed ‘E’, valence felt and expressed ‘V’, arousal felt and expressed ‘A’, and dominance felt and expressed ‘D’) and then summing these four values (|E|+|V|+|A|+|D|).

To assess the degree to which negative emotion predicted enjoyment of the music relative to the other listening appraisal responses, a multiple linear regression was conducted using the Loved rating as the dependent variable. The predictors entered were the 19 listening appraisal items, as well as the GAEL score. The higher the GAEL score, the larger the overall difference between felt and expressed emotion ratings.

Overall, the model explained 82.2% of the variance (see Table 4). GAEL had a significant negative coefficient, meaning that smaller GAEL values are preferred, consistent with previous research (for a review, see [22]). Negative emotion did not make a significant contribution to the model, suggesting that high negative emotion ratings were not associated with low or high Loved ratings, which is not consistent with the above analyses of negative emotion enjoyment. It is possible, however, that both positive and negative relationships may be present but buried in the data, cancelling each other out and, thus leading to an overall non-significant contribution of negative emotion at the pooled level.

**Table 4:** Coefficients for three multiple linear regression models for Loved music ratings.

	Overall Model (Adj R^2^ = .823)		Low Trait Absorption Group Model (Adj R^2^ = .858)		High Trait Absorption Group Model (Adj R^2^ = .844)	
Predictor	B (SE)	β	B (SE)	β	B (SE)	β
(Constant)	0.34 (0.636)		-1.119 (0.924)		1.166 (0.862)	
GAEL	-0.092 (0.044)	-0.075*	-0.074 (0.064)	-0.058	-0.074 (0.058)	-0.062
Familiarity	0.2 (0.05)	0.143*	0.308 (0.066)	0.225*	0.143 (0.075)	0.097
Quality	0.265 (0.058)	0.216*	0.166 (0.068)	0.134*	0.344 (0.094)	0.281*
Felt Strong Emotion	0.261 (0.062)	0.246*	0.144 (0.09)	0.124	0.325 (0.08)	0.329*
Felt Positive Emotion	0.288 (0.066)	0.247*	0.455 (0.09)	0.357*	0.171 (0.088)	0.158
Felt Active Emotion	-0.111 (0.059)	-0.094	-0.11 (0.083)	-0.09	-0.127 (0.078)	-0.111
Felt Dominant Emotion	-0.071 (0.057)	-0.057	-0.171 (0.077)	-0.132*	0.017 (0.079)	0.014
Felt No Emotion	-0.114 (0.054)	-0.102*	-0.043 (0.074)	-0.036	-0.171 (0.073)	-0.161*
Felt Negative Emotion	0.031 (0.049)	0.026	-0.062 (0.066)	-0.047	0.141 (0.07)	0.126*
Felt Sleepy Emotion	-0.108 (0.047)	-0.091*	-0.164 (0.067)	-0.133*	-0.042 (0.063)	-0.037
Felt Submissive Emotion	0.001 (0.056)	0.001	-0.117 (0.088)	-0.066	0.01 (0.069)	0.007
Music Strong Emotion	0.188 (0.066)	0.162*	0.344 (0.091)	0.274*	0.107 (0.09)	0.099
Music Positive Emotion	-0.069 (0.056)	-0.056	-0.1 (0.072)	-0.07	-0.051 (0.076)	-0.046
Music Active Emotion	0.062 (0.058)	0.05	0.053 (0.082)	0.038	0.066 (0.078)	0.059
Music Dominant Emotion	0.017 (0.054)	0.014	0.087 (0.074)	0.069	-0.022 (0.074)	-0.019
Music No Emotion	-0.052 (0.057)	-0.037	-0.043 (0.086)	-0.027	-0.047 (0.073)	-0.038
Music Negative Emotion	0.036 (0.052)	0.03	0.107 (0.072)	0.081	-0.122 (0.073)	-0.111
Music Sleepy Emotion	-0.024 (0.053)	-0.019	0.169 (0.078)	0.128*	-0.143 (0.068)	-0.122*
Music Submissive Emotion	-0.016 (0.051)	-0.012	0.041 (0.075)	0.026	-0.009 (0.067)	-0.007

* *p* < .05; β = standardised coefficient; GAEL = Gap across emotion loci using the |A|+|V|+|D|+|E| scores

All entered variables exhibited tolerance greater than .2, which according to Menard ([43], p. 66) is considered an acceptable level of multicollinearity. The predictor exhibiting the lowest tolerance was ‘I felt strong emotion’ for the Low Trait Absorption model (with a value of 0.203).

To address this, we tested whether absorption accounted for a difference in direction of the contribution of negative emotion to preference by running two additional multiple linear regression analyses, one for the low trait absorption group and one for the high trait absorption group, again using Loved ratings as the dependent variable. The four columns on the right of Table 4 show the coefficients of the two models. Critically, felt negative emotion is a significant positive predictor of preference in the high trait absorption group but not the low trait absorption group, suggesting that participants in the high trait absorption group enjoy negative emotion in music.

## Discussion and Conclusions

This study investigated the relationship between absorption and music preference. Specifically, we sought to compare the influence of trait versus state absorption on music enjoyment, particularly enjoyment of music that evokes negative emotions. First, we examined whether negative emotion evoked by music *could* be enjoyed, and under what circumstances. In line with a growing body of evidence for the ‘paradoxical’ effect of enjoyment of negative emotions in music [20], a subset of our sample reported high enjoyment of self-selected music that was strongly evocative of negative emotions. Crucially, we found that this subgroup of individuals tended to be those with a (trait) propensity to be highly absorbed. This is consistent with previous findings that enjoyment of negative emotions in music is more common among individuals high in trait absorption [17].

Theoretically, this finding can be interpreted according to Schubert’s [21, 44] proposal that the enjoyment of negative emotions in music is made possible through a process of ‘disconnection’ from the unpleasant aspects of the negative emotion. As individuals with high trait absorption are, by definition, more readily able to enter a normative dissociated state, they may be particularly proficient at disconnecting from unpleasant aspects of negative emotion in music and instead, becoming highly immersed in the pleasurable aspects of the music. This may include, for example, the energising properties of music that evokes highly arousing negative emotions. Indeed, in contrast to the argument that the enjoyment of negative emotions in music is restricted to a small set of low arousal emotions such as sadness [19, 45], we found evidence that individuals can report enjoyment of negative emotions with a range of arousal levels. Further research is required to assess the possibility that specific, discrete negative emotions being evoked by music, such as fear and anger, can be enjoyed.

In contrast to trait absorption, which appears particularly important in predicting individual preference for music that evokes negative emotions, we found that state absorption was the primary driver of music enjoyment in the moment. Specifically, participants who entered an absorbed state were more likely to rate their chosen piece of music as highly loved than participants who did not enter an absorbed state. Thus, where trait absorption appears important for determining music preference based on its emotional content, the actual experience of entering an absorbed state in the moment appears to be the more proximal driver of whether a specific music listening experience is enjoyed. Our empirical findings build upon previous qualitative research proposing a link between states of absorption during music listening and enjoyment [3]. Our findings are also consistent with literature on the conceptually related construct of ‘flow’, where states of total immersion in an activity are associated with maximal enjoyment [6].

Finally, in addition to absorption, we examined the role of other participant-rated musical characteristics including quality, familiarity, emotional responses, and Gap Across Emotion Loci (GAEL) in predicting musical preference. In support of the importance of these factors, our model explained a considerable portion of the variance in music enjoyment (82%). Of particular interest was the finding that the degree of correspondence between the emotion expressed and emotion evoked by the music (GAEL) was predictive of music preference. We extended previous work by incorporating two additional emotional variables into the construct of GAEL, strength and dominance, in addition to the two dimensions traditionally used (i.e., valence and arousal). Thus, our findings build upon previous studies (for a review, see [22]) by demonstrating that, in addition to valence and arousal, the extent of the match between the strength and the dominance of emotions expressed and induced by the music is also important in predicting preference.

In terms of methodological contributions, the use of unipolar emotion rating scales rather than traditional bipolar valence scales (negative to positive) allowed us to tease apart the unique contribution of different aspects of emotion on preference. This approach also allowed us to obtain a clearer picture of how negative emotions relate to preference along the theoretically-driven dimensions of emotional arousal, strength, and dominance, without limiting them to specific, discrete emotions such as sadness, grief, anger and fear. As a result, we are able to conclude that felt negative emotion ratings play a significant role in the enjoyment of music for people with a high propensity to be absorbed. Interestingly, unipolar felt positive emotion was found to be a very strong predictor of preference, exerting effects independent of felt negative emotion (in line with [46, 47]). When positive emotions are rated using a bipolar item (positive-negative), simultaneous positive and negative emotions must be somehow melded, diluted or partially ignored to allow adherence to the single scale forced choice response, possibly also diluting an underlying relationship between valence and preference, should one exist. Using the unipolar positive emotion scale, we were able to detect a clear, positive relationship with preference.

In terms of possible alternative explanations for our findings, Eerola and colleagues [19] have argued that the finding of enjoyment of negative emotion in music could be an artefact of sampling (e.g. a tendency to select participants who are likely to enjoy such music). However, the present study made no screening of trait or musical preference characteristics. Furthermore, when considered at the aggregate (total sample) level, negative emotion was not a predictor of enjoyment; relationships between negative emotions and enjoyment were only revealed when examining the subset of individuals with high trait absorption. This provides evidence against the likelihood of sampling bias in our study.

The study adopted a reductionist approach to absorption by applying an established psychometric measure of trait absorption, and direct, quantitative probing of state absorption experiences. Future research could augment this approach by examining more sophisticated measures of both state and trait absorption, as proposed by Herbert [3]. However, our results indicate that the quantitative measurement of state and of trait absorption may provide a meaningful way forward in assessing the relative influence of absorption upon preference. Future approaches may also try another alternative, which is to adapt questions from the trait inventory to instance-based absorption and only use those items where the event under investigation (such as a music listening experience) can be directly translated from the trait absorption item. Also, the present study allowed participants to complete a listening experiment in a location of their choice. Although this web-based approach has several advantages [48], replication under more environmentally controlled conditions may further vindicate the use of ecologically realist settings in future research.

In conclusion, absorption appears to play an important role in how we engage with musical stimuli, with state absorption being related to music preference and trait absorption being related to preference for negative emotions in music. Future research will need to investigate whether these are general principles by examining a range of artistic experiences. Finally, our findings support the use of state and trait measures for explaining distinct aspects of psychologically-mediated behaviours.
